# Treatment with hESC-Derived Myocardial Precursors Improves Cardiac Function after a Myocardial Infarction

**DOI:** 10.1371/journal.pone.0131123

**Published:** 2015-07-31

**Authors:** Jianqin Ye, Meenakshi Gaur, Yan Zhang, Richard E. Sievers, Brandon J. Woods, Julian Aurigui, Harold S. Bernstein, Yerem Yeghiazarians

**Affiliations:** 1 Department of Medicine, University of California San Francisco, San Francisco, California, 94143, United States of America; 2 Cardiovascular Research Institute, University of California San Francisco, San Francisco, California, 94143, United States of America; 3 Eli and Edythe Broad Center of Regeneration Medicine and Stem Cell Research, University of California San Francisco, San Francisco, California, 94143, United States of America; Georgia Regents University, UNITED STATES

## Abstract

**Background:**

We previously reported the generation of a reporter line of human embryonic stem cells (hESCs) with enhanced green fluorescent protein (eGFP) expression driven by the α-myosin heavy chain (αMHC) promoter. The GFP^+^/αMHC^+^ cells derived from this cell line behave as multipotent, human myocardial precursors (hMPs) *in vitro*. In this study, we evaluated the therapeutic effects of GFP^+^/αMHC^+^ cells isolated from the reporter line in a mouse model of myocardial infarction (MI).

**Methods:**

MI was generated in immunodeficient mice. hMPs were injected into murine infarcted hearts under ultrasound guidance at 3 days post-MI. Human fetal skin fibroblasts (hFFs) were injected as control. Cardiac function was evaluated by echocardiography. Infarct size, angiogenesis, apoptosis, cell fate, and teratoma formation were analyzed by immunohistochemical staining.

**Results:**

Compared with control, hMPs resulted in improvement of cardiac function post-MI with smaller infarct size, induced endogenous angiogenesis, and reduced apoptosis of host cardiomyocytes at the peri-infarct zone at 28 days post-MI.

**Conclusion:**

Intramyocardial injection of hMPs improved cardiac function post-MI. The engraftment rate of these cells in the myocardium post-MI was low, suggesting that the majority of effect occurs via paracrine mechanisms.

## Introduction

We previously generated a reporter line of human embryonic stem cells (hESCs) with enhanced green fluorescent protein (eGFP) expression driven by an α-myosin heavy chain (αMHC) promoter.[1] GFP^+^/αMHC^+^ cells isolated from this line at the onset of GFP expression (day 8 of directed cardiac differentiation) by fluorescence-activated cell sorting (FACS) have been shown to give rise to atrial and ventricular cardiomyocytes (CMs) and Na/K hyperpolarization-activated cyclic nucleotide-gated channel 4 (HCN4^+^) specialized conduction cells *in vitro* and *in vivo*.[1] Using this reporter line, we isolated early human myocardial precursors expressing Nkx2-5, but not expressing mature CM markers, such as cardiac troponin I (TnI) or myosin light chain, and we therefore refer to these cells as human myocardial precursors (hMPs).[1] Previous studies have shown that undifferentiated hESCs form teratomas,[2,3] while fully differentiated hESC-derived CMs provide limited functional benefit *in vivo*.[3,4] Therefore, it was our hypothesis that partially differentiated hMPs would demonstrate engraftment and functional benefit post-MI superior to what historically has been seen in studies with hESC-derived CMs including our own,[5] and be less likely to result in teratoma formation compared with undifferentiated hESCs.

In this report, we used a myocardial infarction (MI) model in immunodeficient mice as previously reported,[5] injected hMPs into infarcted myocardium at 3 days post-MI by closed-chest ultrasound-guided injection,[6] and compared the functional and tissue effects to human fetal skin fibroblasts (hFFs) as control. We found that hMPs improved cardiac function, limited infarct size, induced endogenous angiogenesis, and reduced apoptosis of host CMs at 28 days post-MI compared with hFFs. However, the engraftment rate of hMPs was not significantly different compared with hESC-derived CMs differentiated in culture for 21 days.[5] No teratoma was noted in injected hearts by hematoxylin and eosin staining at 28 days post-MI.

## Materials and Methods

### Preparation of GFP/αMHC-expressing hMPs and hFFs

All procedures with hESCs were approved by the Human Gamete, Embryo and Stem Cell Research Committee of University of California San Francisco (Approval number: 10–04745). The parent H9 hESC line (WA09) was purchased from WiCell Research Institute (Wisconsin). A hESC line with eGFP expression driven by an αMHC promoter was generated and maintained as previously described.[1] Differentiation was initiated by human embryoid body (hEB) formation in suspension. Briefly, colonies of hESCs were dissociated into small clusters by exposure to Collagenase IV (Sigma-Aldrich), then allowed to differentiate in a medium comprised of Knockout DMEM (Invitrogen) supplemented with 20% Defined Fetal Bovine Serum (Hyclone), 2mM glutamine, 0.1mM non-essential amino acids, and 0.1mM β-mercaptoethanol. After 7 days in suspension, hEBs were attached to gelatin-coated 12-well culture plates and allowed to differentiate for an additional 7 days [4,7]. The differentiating reporter cells start expressing GFP on day 8 in suspension. On day 14, hEB were treated with 10μM ROCK inhibitor (Y-27632; Calbiochem) in differentiation medium overnight. hEBs were dissociated with TrypLE Express (Invitrogen) to generate single cell suspensions, stained with propidium iodide to distinguish between live and dead cells, and sorted on the basis of GFP expression using a FACSAria (Becton Dickinson). The sorted GFP^+^/αMHC^+^ cells were re-suspended in MEF-conditioned medium at a concentration of 10^5^/10μl for intramyocardial injection (5μl/injection, 2 injections/heart). The viability of sorted cells was assessed by Trypan blue staining at 90%.

hFFs (CCL110, ATCC) were cultured in Modified Eagle’s Medium with Earl’s BSS containing 10% FBS, 1x non-essential amino acids as recommended by the provider. hFFs were trypsinized and re-suspended in MEF conditioned medium at a concentration of 10^5^/10μl for intramyocardial injection (5ul/injection, 2 injections/heart).[5]

### Animals and groups

All experiments with twelve week-old, female SCID-Beige (CB17SC-F C.B-*Igh-1* ^*b*^/IcrTac-*Prkdc* ^*scid*^, Taconic) mice were approved by the Institutional Animal Care and Use Committee of University of California San Francisco (Appproval number: AN089562). Mice were injected with hESC-derived hMPs (n = 19; active treatment) or hFFs (n = 8; control).

### Myocardial infarction and echocardiography

MI was surgically induced as previously described.[5,6] Briefly, animals underwent total permanent ligation of the left anterior descending artery to create a MI at 50% of the length of the heart from the anterior-inferior edge of the left atrium to the apex.

Echocardiography was performed under isofluorane anesthesia with the use of a Vevo660 (VisualSonics) equipped with a 30 MHz transducer as previously described. [5,6] Echocardiograms were obtained at baseline, 2 days post-MI (before injection), and at day 28 post-MI (25 days post-injection). We measured left ventricular (LV) ejection fraction (LVEF), end-systolic volume (ESV), end-diastolic volume (EDV), and wall thickness. Wall thickness was measured at the apical anterior wall (infarct wall thickness) and at the mid-anterior segment (peri-infarct wall thickness) separately on the parasternal long-axis view. Posterior wall thickness was measured at the papillary muscle level. Three cycles were measured for each assessment and average values were reported. Acquisition of Echocardiographic images and data analysis was performed in a blinded manner.

### Ultrasound-guided closed-chest Injections

All injected cells were suspended in differentiation medium as described above. Animals underwent ultrasound-guided injection of hMPs or hFFs at 3 days post-MI as previously described. [5,6,8] Each heart was injected at 3 days post-MI with 10^5^ cells in 10 μl of MEF conditioned medium, divided into two 5 μl injections into the anterior wall.

We have developed an ultrasound guided closed-chest injection technique into the rodent heart post-MI and using this method, the injected cells are delivered to the peri-infarct regions under direct visual guidance, and also allow us to inject the cells at 3 days post-MI without redo thoracotomy which is associated with a higher animal mortality.[8] Injecting cells into infarcted hearts at 3 days post-MI is a more clinically relevant timeframe. [5,6,9]

### Tissue analysis

Tissues were analyzed by two blinded reviewers as previously described.[5] Mice were sacrificed 28 days post-MI (25 days post-injection). The hearts were arrested in diastole with KCl, perfused and fixed with 10% formalin, embedded in paraffin, and cut into 5 mm sections.

Several levels of sections from each group were stained with hematoxylin and eosin and analyzed for the presence of teratomas as previously described.[5]

Infarct size was measured histologically as previously described.[5,6,10] Briefly, the sections from mid-ventricular level (mid-papillary) were stained using a Masson’s Trichrome 2000 Staining kit (American Master Tech Scientific, Inc. Lodi, CA). Sections were examined with a Nikon Eclipse E800 microscope using a 1x objective and analyzed using Openlab software (Improvision, Lexington, MA). Infarct size was measured by calculating the percentage of infarct zone area in the LV using ImagePro software (MediaCybernetics; Bethesda, MD, USA) as described previously.[5,6]

Apoptosis was assessed using terminal deoxynucleotidyl transferase dUTP nick end labeling (TUNEL) performed with ApopTag Plus Peroxidase *In Situ* Apoptosis Detection Kit (Chemicon, Temecula, CA) according to manufacture’s protocol. To detect cardiac TnI, sections from mid-ventricular level were treated with denature solution (Biocare), blocked with Rodent Block M, and incubated with mouse anti-TnI (ab19615, Abcom, Cambridge, MA, USA). The mouse-on-mouse alkaline phosphatase polymer (Biocare) was used as secondary antibody. Vulcan Fast Red Chromogen kit was used for color development, and sections were counterstained with hematoxylin. TUNEL-positive CMs were defined by the presence of DAB nuclear staining completely surrounded by cytoplasmic TnI staining.

Proliferating CMs were detected using anti-Ki-67 (DAKO; Carpinteria, CA, USA) counterstained for TnI. Proliferating CMs were defined by the presence of Ki-67^+^ nuclei completely surrounded by cytoplasmic TnI staining. Apoptotic and proliferating CMs were quantified as the number of positive cells in five high-power fields (HPF) examined for each of the relevant myocardial zones relative to the infarct, i.e., infarct zone (IZ), peri-infarct zone (PZ) and remote zone (RZ).

### Immunohistochemical detection of retained hMPs

At days 14 and 28 post-MI, injected GFP^+^/αMHC^+^ hMPs were detected by immunostaining using chicken anti-GFP primary antibody (1:100, Aves Labs) and horseradish peroxidase-labeled goat anti-chicken secondary antibody (1:300, Aves labs), with subsequent detection with DAB reagent (Biocare). Antigen retrieval was facilitated by incubating sections with proteinase K before staining.

The fate of injected GFP^+^/αMHC^+^ hMPs also was determined by immunofluorescence staining of human cardiac TnI. Sections were incubated with rabbit anti-hcTnI antibody (Abcam) following antigen retrieval by heat-induced epitope retrieval in sodium citrate, pH 6.0. Alexa 546-conjugated donkey anti-rabbit (Invitrogen) was used for detection. Slides were mounted with ProLong Gold antifade reagent with DAPI. Sections were viewed and analyzed using a Nikon Eclipse E800 fluorescence microscope and Openlab software.

### Statistical analysis

One way ANOVA with Fisher’s post hoc test was used to analyze the difference among multiple groups. Student’s *t* test was used to analyze differences between two groups. Values were expressed as mean±SD unless otherwise specified, with P<0.05 considered significant. SPSS 15.0 software was used to conduct all statistical analysis.

## Results

### Injected hMPs improve cardiac function and reduce infarct size

**Fig 1:**
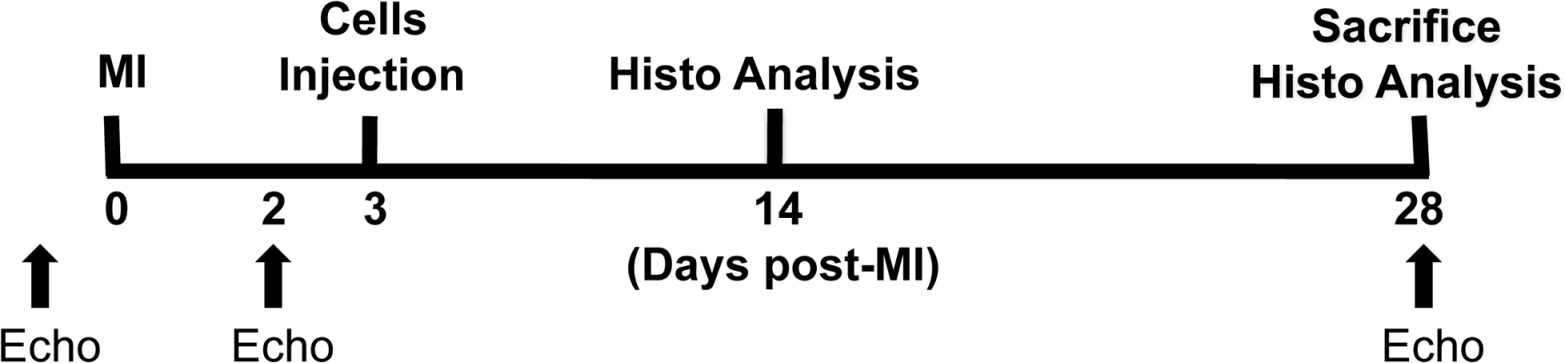
Schematic representation of experiment design. MI: myocardial infarction; Echo: echocardiography; Histo: histological.

**Fig 2:**
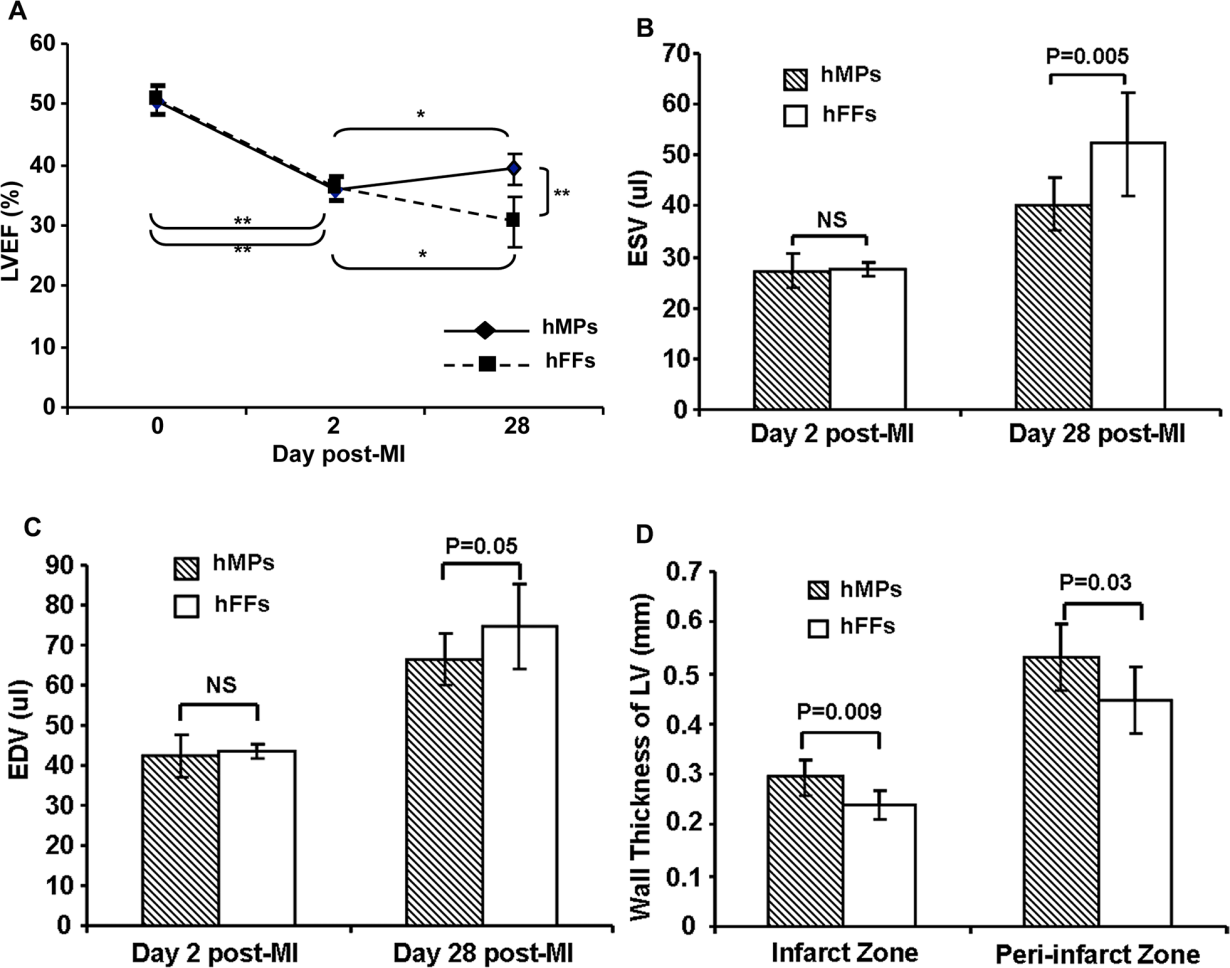
Injected hMPs improve cardiac function at day 28 post-MI. (A) Left ventricular ejection fraction (LVEF) with hESC-derived hMPs injection improved compared to control. Each line represents the mean of one group, * P<0.03; ** p<0.0001. (B) End-systolic volume (ESV) and (C) end-diastolic volume (EDV) were measured over time, hearts injected with hESC-derived hMPs show sustained decreases in the volumes over time, with less evidence of dilatation compared with control. (D) Wall thickness in the peri-infarct zone (PZ) measured echocardiographically was thicker in hESC-derived hMPs injection group compared to control. hMPs: n = 10; hFFs: n = 5.

**Fig 3:**
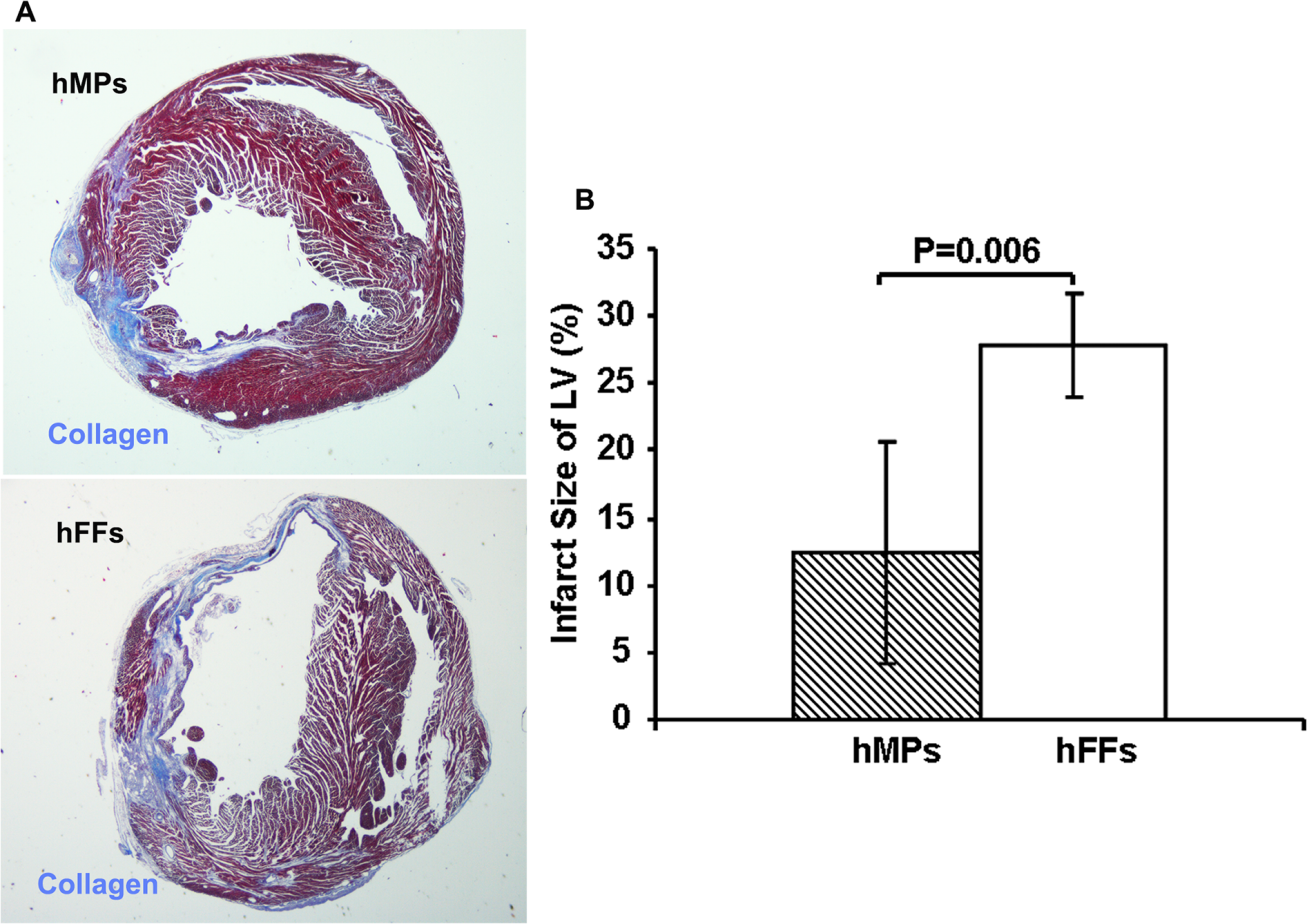
Injected hMPs reduce infarct size at day 28 post-MI. (A) Micrograph of section of hearts stained with Masson’s trichrome. Collagen stained as blue; myocardium stained as dark red. (B) Mice treated with hESC-derived hMPs (n = 8) had smaller infarct sizes compared to those treated with hFFs (n = 4, P<0.006).

LVEF was uniformly and significantly reduced from about 50.7% in both groups before MI to 35.9% (hMPs group) and 36.2% (hFFs group) at 2 days post-MI (P<0.0001), with no significant differences between two groups (Figs 1 and 2A). At 28 days post-MI, LVEF was improved significantly with injection of hMPs (39.3±2.7%), while LVEF continued to decline with injection of hFFs (30.7±4.1%) and the resultant LVEF of hMPs-injected group was significantly higher than that of hFFs-injected group (P*<*0.0001, Figs 1 and 2A). At 28 days post-MI, there was also less LV dilatation in the hMP-injected group (ESV: 40.4±5.2 μl; EDV: 66.4±6.4 μl) compared with the hFF-injected group (ESV: 52.3±10.1 μl, P = 0.005; EDV: 74.9±10.6 μl, P = 0.05, Fig 2B and 2C). Furthermore, there was significantly less wall thinning of the LV PZ in the hMP-injected group (0.53±0.07mm) compared with the hFF-injected group (0.45±0.07mm, p = 0.03, Fig 2D). The hMP-injected hearts also showed significantly smaller infarct size (12.44±8.29% of LV) compared to the hearts injected with hFFs (27.82±3.96% of LV) at 28 days post-MI (P *=* 0.012; Figs 1 and 3).

### Fate of implanted hMPs

**Fig 4:**
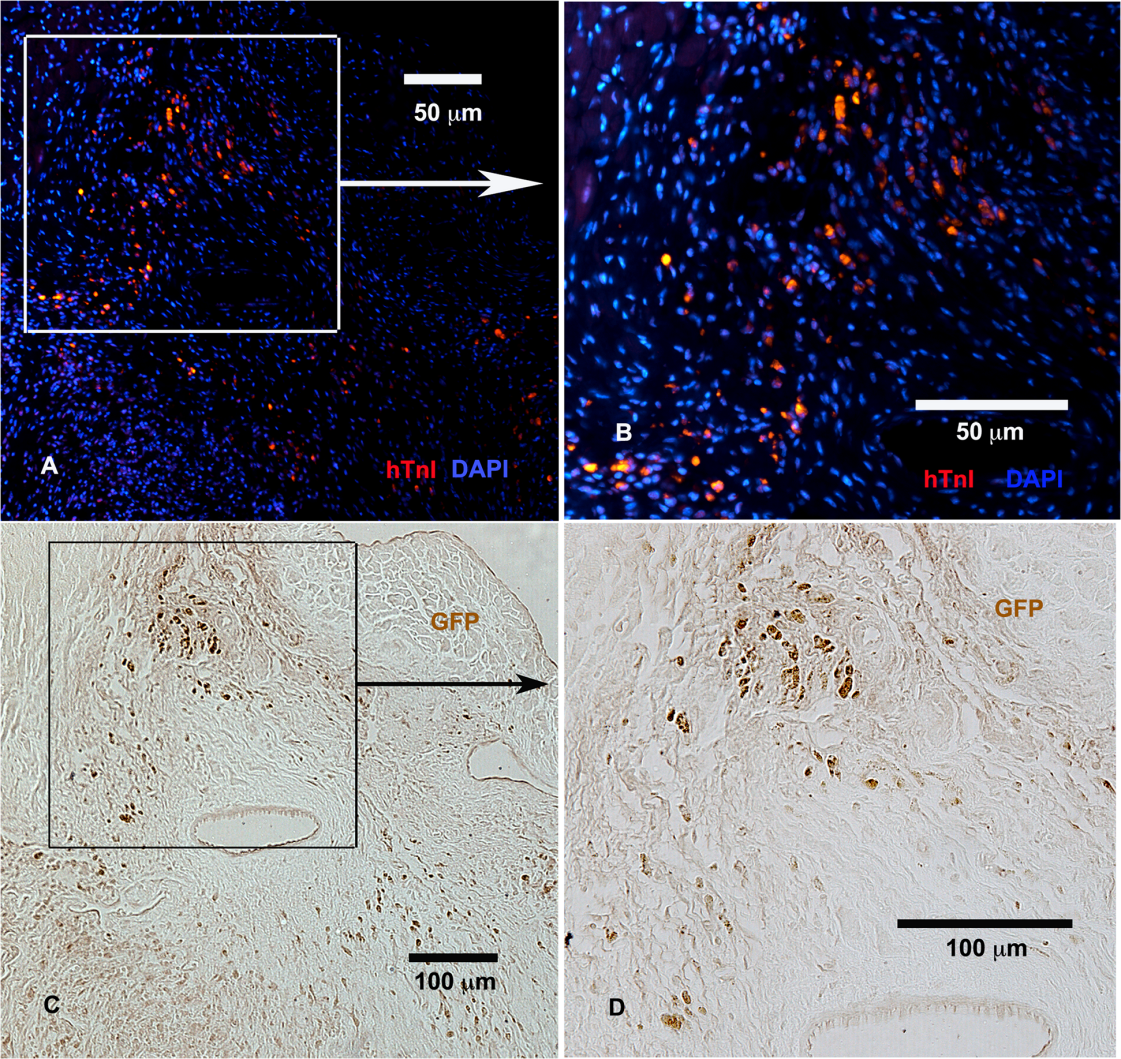
Injected hMPs can be detected in recipient mouse hearts at 14 days post-MI. The surviving injected cells expressed human cardiac Troponin I (hTnI, A, B) and green fluorescence protein (GFP, C, D) in the peri-infarct zone (PZ, n = 4). Nuclei were stained with DAPI (A, B).

Previous studies including ours[5] have shown very low engraftment rates for hESC-derived CMs. As such, we investigated the fate of implanted hMPs at early (14 days post-MI) and late (28 days post-MI) time points. Immunohistochemical evaluation of tissue sections both at the injection sites and throughout the recipient hearts showed few retained human cTnI^+^ (n = 4, Fig 4A and 4B) or GFP^+^ cells (n = 4, Fig 4C and 4D) at 14 days post-MI and none at 28 days (n = 4, data not shown).

We also examined whether hMPs could differentiate into endothelial and smooth muscle cells by co-staining GFP^+^ cells for CD31 and smooth muscle α-actin (SMA). However, no GFP^+^/SMA^+^ or GFP^+^/CD31^+^ cells were detected by immunohistochemical staining at day 28 post-MI (data not shown). This suggested that hMPs did not differentiate into endothelial and smooth muscle cells *in vivo*, consistent with our previous report that hMPs only differentiate into CMs and conduction cells.[1]

Detailed analysis of hematoxylin and eosin stained sections showed no evidence of teratoma formation in the heart for any of the experimental groups at 28 days post-MI and no evidence of teratoma formation in lung, liver, spleen by gross inspection (data not shown).

### Injected hMPs Increased angiogenesis

**Fig 5:**
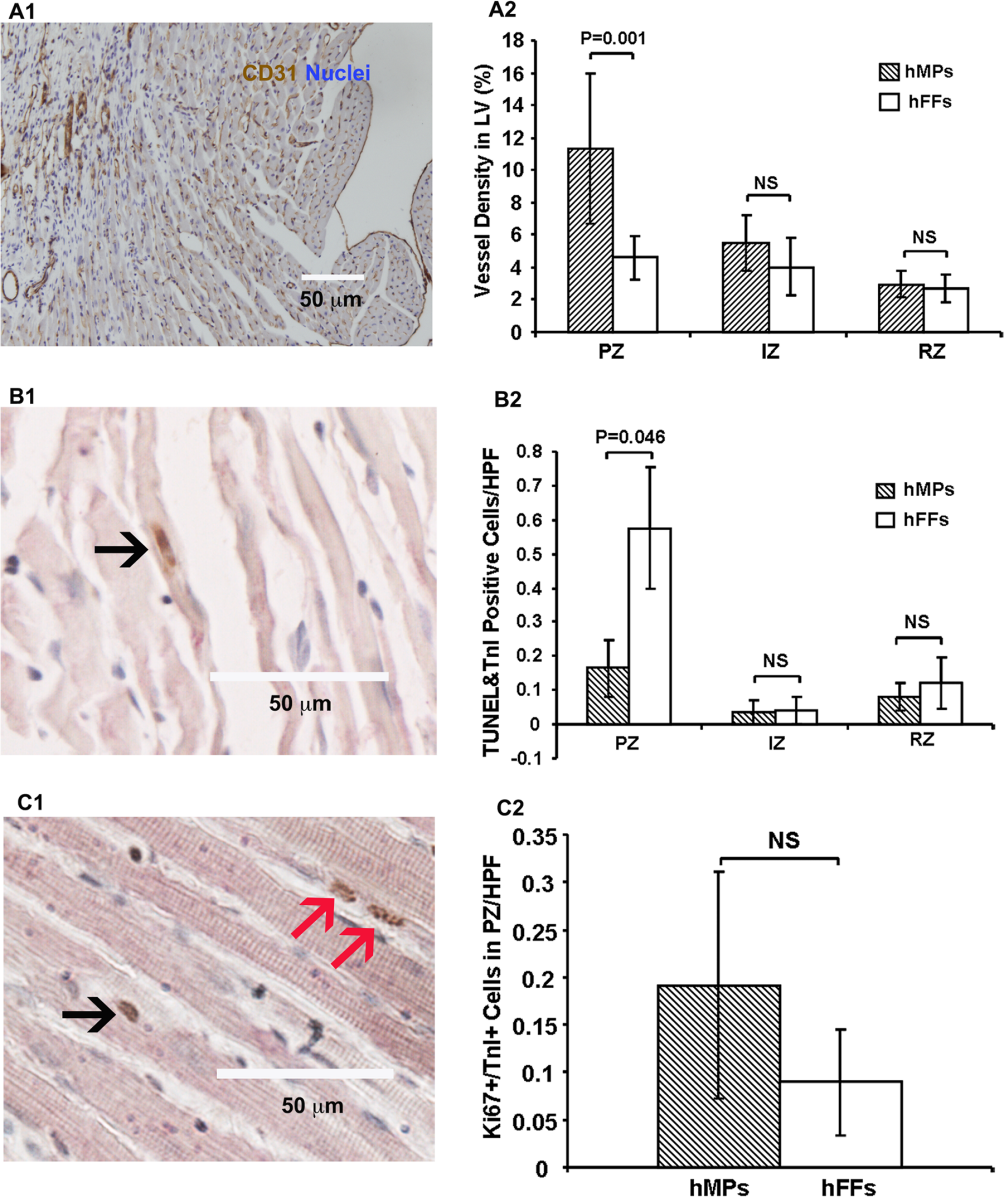
Injected hMPs induce angiogenesis, reduce host CM apoptosis, but do not increase CM proliferation at 28 days post-MI. (A1) Image shows CD31^+^ cells. (A2) hMP injection (n = 7) results in a significantly increased CD31^+^ cells in the peri-infarct zone (PZ) (P = 0.001), but not in the infarct zone (IZ) and remote zone (RZ) compared to hFF injectin (n = 8). (B1) Typical image showing TUNEL^+^/Troponin I^+^ cells (black arrow). (B2) hMP injection (n = 7) results in a significant reduction of TUNEL^+^/Troponin I^+^ cells in PZ (P = 0.046), but not in IZ and RZ compared to the hFF injectin (n = 8). Data are shown as mean±SEM. (C1) Typical image showing Ki67^+^/Troponin I^+^ cells, black arrows, apoptotic CMs; red arrows, apoptotic cells that are not CMs. (C2) The number of Ki67^+^/Troponin I^+^ cells did not differ between hMP (n = 6) and hFF (n = 5) treated groups. Data are shown as mean±SEM. TnI: troponin I; TUNEL: terminal deoxynucleotidyl transferase dUTP nick end labeling; CMs: cardiomyocytes; HPF: high power field (20 x magnification); hFFs: human fetal fibroblasts; NS: no significant difference.

To determine the effects of hMP transplantation on endogenous angiogenesis, we assessed capillary density at day 28 post-MI. Capillaries at the mid-papillary level of LV were stained with antibody against CD31. The results showed that the density of capillary was significantly increased in the hMP-injected group (11.31±4.60% of LV) compared to the hFF-injected group (4.59±3.44% of LV; P = 0.001) in the PZ, although there was no difference at the IZ and RZ (Fig 5A).

### Injected hMPs inhibit native CM apoptosis but have no effect on CM proliferation

To evaluate the effects of hMPs on host CM apoptosis, we used TUNEL and TnI co-staining in the hearts 28 days post-MI. hMP injection resulted in significant reduction in the number of TUNEL^+^/TnI^+^ cells in the PZ compared to hFF injection (0.16±0.08 vs. 0.57±0.17/HPF, P<0.046) (Fig 5B). The number of TUNEL^+^/TnI^+^ cells did not differ between hMP- and hFF-injected groups in the IZ and RZ (Fig 5B).

To evaluate the effects of hMP therapy on the proliferation of host CMs, we examined Ki67 expression in host CMs at 28 days post-MI. The results showed that there was no significant difference in host Ki67^+^/TnI^+^ CM in PZ between hMP and hFF groups (hMPs: 0.19±0.12; hFFs: 0.09±0.06/HPF, Fig 5C).

## Discussion

In this study, we used hMPs derived from an αMHC-GFP hESC reporter line to treat the ischemic heart in a mouse MI model. The major findings of this report are: 1) injection of hMPs improved cardiac function, and limited infarct size and deleterious LV remodeling at 28 days post-MI compared to hFF-injection; 2) hMP treatment post-MI induced endogenous angiogenesis and decreased host CM apoptosis at 28 days post-MI; 3) the retention of hMPs after injection into the heart was very low and there was no CM differentiation from the injected cells; and 4) hMP therapy did not result in teratoma formation at 28 days of follow-up in the immunodeficient mouse MI model.

We hypothesized that injecting hMPs might result in greater cell retention and more *in vivo* differentiation into mature CMs. However, we did not detect hMP differentiation into mature CMs *in vivo* and most of the injected cells disappeared after 2 weeks post-MI, which is similar to injecting hCMs in a previous report by us.[5] Notably, a recent report [11] suggested that it might take 24 weeks for injected hESC-derived CMs to differentiate into mature CMs *in vivo*; however, our findings of low cell retention raises doubts about this hypothesis. Our results suggest that the ischemic deleterious environmental in the injured myocardium make survival of implanted cells difficult for any cell type [4]. Ongoing research using biodegradable scaffolds to improve survival and engraftment of stem cells in the myocardium post-MI is currently being pursued. [12,13]. In fact, recent studies in non-human primates suggest that significant cell retention and neo-muscularization using hESC-derived CMs treated with a pro-survival cocktail is possible [14].

Despite the low cell retention, injection of hMPs resulted in an increase in capillary bed area and a decrease in host CM apoptosis compared to injection of hFFs. These results are in agreement with previous reports including ours,[5,6,15] suggesting that many different cell types appear to improve cardiac function post-MI but that the retention rate of all injected cells is low and that the majority of the benefit is likely via paracrine effects [16]. These are acknowledged as the primary mechanisms by which mesenchymal stem cells (MSCs)[17] and MSC-derived ‘cardiopoietic’ cells[18] achieve their restorative effects in clinical studies.

Animal research and clinical trials have demonstrated that cardiac function might be improved by stem cell therapy.[16,19] However, in the majority of the reported studies, the number of differentiated CMs from injected stem cells is too small to explain the observed improvements in cardiac function.[16,19] Current hypotheses to explain the improvements include that the transplanted cells release soluble growth factors and cytokines that enhance angiogenesis, reduce apoptosis of existing CMs, promote CM re-entry into the cell cycle,[20–23] and recruit endogenous cardiac progenitors.[24,25] These beneficial effects are believed to contribute to limiting or reducing infarct size and improving cardiac function. There are reports that injected hESC-derived hCMs continue to secrete collagen IV, XVIII and fibronectin *in vivo*,[11] and injected murine ESC-derive CMs secrete IL-1a, IL-6, tumor necrosis factor-b, and oncostatin M,[26] however, the specific paracrine factors responsible for the effects induced by hESC-derived cells remain to be fully elucidated.[27]

Undifferentiated hESCs are known to produce teratomas when introduced into a physiological environment.[2,3,28] This is a primary safety liability of pluripotent stem cell therapy. Here we isolated hMPs from differentiating hESCs and injected these cells into injured mouse hearts, and did not detect teratomas at 28 days post-MI. This suggests that selection of a multipotent CM precursor may provide a safer approach to cardiac cell therapy.

In conclusion, compared to our previous work,[5] we observed no significant benefit to cell retention and cardiac function by injecting hMPs compared with hCMs. The low rate of cell engraftment observed with fully differentiated hCMs was not overcome by using partially differentiated hMPs. Ongoing research using pro-survival cocktails and biodegradable scaffolds should lead to improved cell retention and survival, thereby facilitating the efficient and durable paracrine effects of these cells post-MI [4,12,13].
